# Magnonic key based on skyrmion clusters

**DOI:** 10.1038/s41598-021-02285-0

**Published:** 2021-11-26

**Authors:** E. Saavedra, F. Tejo, N. Vidal-Silva, J. Escrig

**Affiliations:** 1 Departamento de Física, Universidad de Santiago de Chile, Avda. Víctor Jara 3493, 9170124 Santiago, Chile; 2 Center for the Development of Nanoscience and Nanotechnology, Avda. Libertador Bernardo O’Higgins 3363, 9170124 Santiago, Chile; 3 Instituto de Ciencia de Materiales de Madrid, CSIC, Cantoblanco, 28049 Madrid Spain; 4 Departamento de Ciencias Físicas, Universidad de La Frontera, Casilla 54-D, 4811186 Temuco, Chile

**Keywords:** Materials science, Nanoscience and technology, Physics

## Abstract

In this work, we report the excitation of spin-waves modes in skyrmion clusters hosted in Co/Pt nanodots by applying an in-plane magnetic field pulse. The direction at which the magnetic field is applied enables the excitation of five main spin-waves modes that are understood in terms of only azimuthal-like modes, which are shown to be strongly dependent on the number of skyrmions stabilized in the system. This feature converts the present system in a mechanism to activate and suppress a set of given modes, which in turn we propose to be utilized as a magnonic key based skyrmion cluster. Our results could be useful in manufacturing potential magnonic logic devices based in skyrmionic textures.

## Introduction

Nontrivial topological magnetic textures are promising elements for potential spintronic^1^, magnonic^2^ and logic devices^3,4^. Skyrmions have been proposed to be an essential ingredient for achieving such a purpose due to their extraordinary properties related to their topologically nontrivial nature^5–7^. The stabilization of skyrmion textures stems from the distinct energy contributions in the system, being the main of them the Dzyaloshinskii–Moriya Interaction (DMI)^8,9^, which is a manifestation of the spin-orbit coupling (SOC) and the breaking of inversion symmetry. In this regard, non-centrosymmetric crystals possess an intrinsic DMI related to the crystallographic arrangement of atoms^7^. On the other hand, multilayer systems can also have DMI induced by the interface between a ferromagnetic layer and a heavy metal with strong SOC, as the case of Co/Pt^10,11^. The interfacial DMI promotes the apparition of Neel skyrmions, which are the focus of this work, and they are characterized by its non-zero radial component in the magnetization vector^12^. In this context, recent experimental studies have shown the possibility of stabilizing skyrmions in multilayer FM/HM stacks^13,14^.

From a fundamental point of view, skyrmions are interesting because, in extended systems, they present topological protection, making them robust under thermal fluctuation, disorder and impurities^15^. The knowledge that we have today about these properties has mainly been possible from the study of isolated skyrmions, which do not interact with other magnetic textures, allowing us to know the fundamental physics that govern their behavior in the absence of other interactions. However, and despite the efforts made in understanding the stability and dynamics of isolated skyrmions, numerous experimental studies have shown that skyrmions spontaneously stabilize in groups, which are called skyrmion clusters^16–19^. To date, skyrmions clusters have been observed in different helimagnetic materials using experimental techniques such as Lorentz Transmission Electron Microscopy (LTEM)^20–23^, Off-axis Electron Holography (EH)^24^, and Cryo-LTEM^25^. One of the relevant aspects that these experiments have in common is that they have shown the possibility of stabilizing skyrmion clusters at room temperature, opening the opportunity for a new exploration of their fundamental properties and possible applications.

One of the highlighted properties that skyrmions clusters possess corresponds to the excitation of spin-waves modes or magnon states. When applying an out-of-plane magnetic pulse, and due to the symmetry of the skyrmion configuration, it is possible to excite the called *breathing mode*^26,27^, which emerge from the periodic contraction and expansion of the skyrmion radius. On the other hand, the application of an in-plane microwave magnetic pulse implies that breathing modes are no longer activated. Instead, azimuthal spin waves modes are excited^28–31^, including the gyrotropic low energy mode. Azimuthal spin waves modes in cylindrical nanodots can be considered as channeling waves along the circular Domain Wall (DW) intrinsically defined by the skyrmion edge (where the out-of-plane magnetization is nearly zero)^32,33^. Consequently, the gyrotropic mode emerges from the hybridization between the skyrmion’s core gyration and the channeling waves^34^, similar to the vortex case^35^. On the other hand, the azimuthal next higher energy modes (hereafter just azimuthal modes with azimuthal index $m = \pm 1$) emerge from the spin excitation located at the skyrmion DW or the nanodot edge, which propagate along counter-clockwise (CCW) or clockwise (CW) direction, and that have also been demonstrated to have nonreciprocity due to the presence of the DMI^32^. Both the skyrmion DW and the nanodot edge act as nanochannels in such a way that these modes behave like spin waves propagating in a magnetic texture background weakly interacting with the skyrmion core. Additionally, the main difference between both azimuthal and gyrotropic modes excited with an in-plane magnetic pulse, is the frequency at which they are excited: the acoustic modes correspond to the gyrotropic ones, and, in contrast, higher resonance frequencies correspond to the azimuthal modes. Thus, while gyrotropic modes emerge from excitation of spins located at the nanodot center, azimuthal modes come from the additional ring-like spin excitations located at the edge of the sample and/or the skyrmion edge, which explains the higher frequency of them^31,36^. Higher order azimuthal modes ($\vert m \vert >1$) might emerge from a non-negligible interaction between the skyrmion core gyration and azimuthal CW or CCW waves^34,35^.

Despite of the excitation of spin-wave modes in skyrmion clusters has been previously studied by applying an out-of-plane magnetic pulse^28,37^, the excitation of spin-wave modes utilizing an in-plane magnetic field pulse has not been addressed in detail so far. In fact, there are just a few works where the dynamical response of these structures has been studied^28^, so that a systematic study is still lacking. This would allow a comprehensive understanding of such systems and eventually pushes the current knowledge on the dynamics of skyrmion clusters to the frontiers.

Following these ideas, our work focuses on provides an alternative way of controlling the spin-wave modes by applying an in-plane magnetic field pulse, which might be used as a switch on/off, or in other words, a magnonic key. A potential magnonic key should be thought to operate by the controlled presence or absence of certain spin-wave modes, in a similar way as in a reconfigurable magnonic crystal the presence or absence of a bandgap could be considered as a switch on/off^38^. The versatility of a magnonic key should also be a parameter to be considered. In specific, it is desired that, for instance, the well working of this device depends on the application of an easily managed external excitation, such that the application of a magnetic field pulse in the range of microwave. By means of micromagnetic simulations, we systematically determined the main resonance peaks and the associated spin-wave modes in the system, which provides a first approach to skyrmion clusters dynamics based on in-plane excitations.

**Figure 1:**
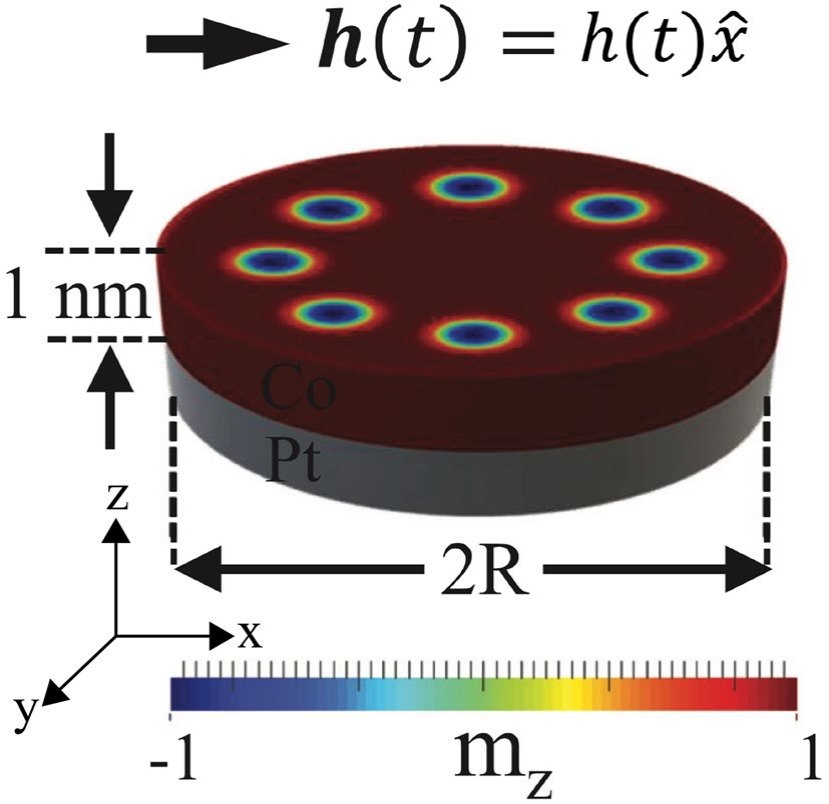
Schematic representation of the system studied.

## Micromagnetic simulations

To study the dynamic response of skyrmion clusters when applying an in-plane magnetic field pulse, we perform micromagnetic simulations using the OOMMF code^39^ with the interfacial DMI extension^12^, enabling the formation of Neel skyrmions. We consider a 1 nm-thick cobalt dot of radius *R* with perpendicular magnetic anisotropy on a Pt substrate inducing DMI, as schematically shown in Fig. 1. This specific system has been proven to host isolated skyrmions as well as a skyrmion cluster configuration^16^. The nanodot radius considered here is $R = 75$ nm. However, we also studied different sizes whose results can be found in the supplementary information. Finally, the system is discretized in cubic cells of 1 $\hbox {nm}^3$. In the following, we detail the procedure to obtain the dynamical properties of the system.

**Figure 2:**
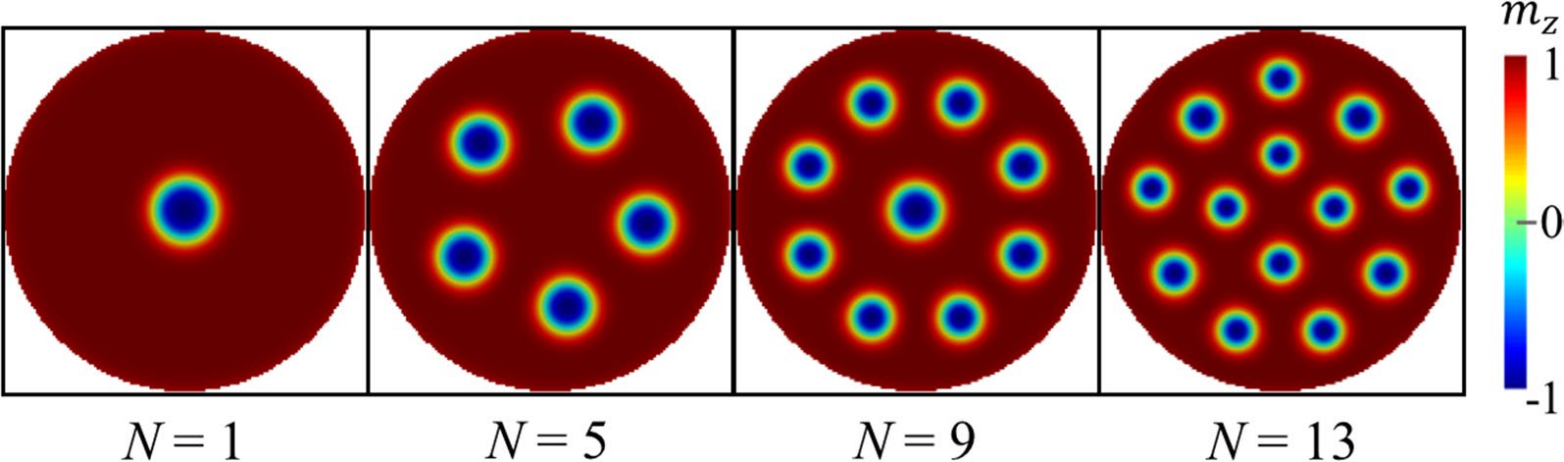
Metastable magnetic states obtained from the minimization energy procedure for the cases $N=1, 5, 9,$ and 13. The number of skyrmions hosted in the nanodot are a consequence of the magnetic parameters chosen here.

### Metastable states

The first step is to stabilize a determined number of skyrmions within the nanodot. To do that, we artificially impose *N*-interacting skyrmions as an initial state, where *N* corresponds to the number of skyrmions in the nanostructure, and which we considered to range from $N=1$ until $N=14$ because larger values of *N* no longer stabilizes a skyrmion cluster structure (see supplementary information for further details on it). Next, we let the system relax by numerically minimizing the following energy functional:

1 $$E_m[{\mathbf {m}}]= \int {\mathrm{d}} V\left [A\sum \limits_{ix,y,z}\left( \nabla m_{i}\right) ^2 + D(m_z\nabla \cdot {\mathbf {m}}-({\mathbf {m}}\cdot \nabla )m_z)-{{K}_{u}} m_{z}^{2}-\frac{M_s}{2}\mu _0{\mathbf {m}} \cdot {\mathbf {H}}_d\right ],$$

The magnetic energy in Eq. (1) comprises the exchange interaction with stiffness constant *A*, Dzyaloshinskii–Moriya energy with the constant *D*, uniaxial magnetic anisotropy parametrized by the anisotropy constant $K_u$, and the dipolar energy expressed in terms of the magnetostatic field ${\mathbf {H}}_d$, respectively; ${\mathbf {m}}={\mathbf {M}}/M_s$ stands for the normalized magnetization, $M_s$ is the saturation magnetization, and $\mu _0$ the vacuum susceptibility. For all our calculations, the magnetic parameters we used are^40^ $M_s=580\times 10^{3}$ A/m, $A = 15\times 10^{-12}$ J/m, $K_u=0.7\times 10^{6}$ J/$\hbox {m}^3$ and $D=3.0\times 10^{-3}$ J/$\hbox {m}^2$. These values have been widely used in previous works^12,41^ for modelling Co/Pt multilayer nanodots and have been chosen because guarantees the stabilization of skyrmion textures as metastable states, which is one of the main focuses of the present work. Additionally, studies based on first principles calculations have reported a $D\sim 3$ mJ/$^2$, whose contribution is predominant on the spins of the interfacial layer of Co adjacent to Pt^11^. The way that the Co/Pt interface is incorporated to our micromagnetic simulations is through modeling it as a relatively thick layer of a magnetic material with a computational cell in which all the magnetic energy is characterized by the parameters *A*, *D*, and $K_u$ in Eq. (1)^12^. We repeated the minimization energy procedure for each value of *N*. Also, different values of the Gilbert damping $\alpha$ were explored in Supplementary Material (Figs. S6, S7) finding no substantial changes on the reported behavior. The results obtained for selected values of *N* are shown in Fig. 2 where we display the metastable magnetic states as a function of the number of skyrmions stabilized in the system (distinct values of *N* can be found in Supplementary Material). Importantly, we identify the cases for $5< N < 11$ where the skyrmion–skyrmion interaction and the edge potential forces the system to locate a skyrmion at the central region. We interpret this behavior in terms of the competition between the two mentioned energies. On the one side, the edge potential pushes the skyrmions towards the nanodot center, while the skyrmion–skyrmion interaction promotes repulsion between them. As a result, the skyrmions accommodate as depicted in Fig. 2. Finally, for the rest of N the central region has no skyrmion states. This eventually has consequences on the dynamical response. It is important to emphasize that our simulations are performed in the absence of defects in the system because otherwise, the skyrmions might be pinned in different sites if the localized pinning potential is stronger than the corresponding competition between the edge potential and the skyrmion–skyrmion interaction, which are both responsible for the reported skyrmion arrangement in these systems. Indeed, although a previous study showed that there are several configurations possible as metastable states in these systems^16^, we have chosen those that correspond to the lowest energy. Nevertheless, we have confirmed that by modeling the presence of grains with a random-localized magnetic anisotropy or an eventual rotation of the skyrmion arrangement due to defects or impurities, our results presented below do not change significantly.

### Dynamical response

Once we have obtained the metastable states, we perturb them to excite the spin-wave modes by applying an in-plane microwave field ${\mathbf {h}}(t) = h(t){\hat{\mathbf {x}}}$. For completeness, we have also performed micromagnetic simulations by applying the microwave field with an angle of $45^{\circ }$ and $90^{\circ }$ with respect to the x-axis, finding no differences with the presented case (see supplementary material). The dynamical response is obtained by numerically solving the Landau–Lifshitz–Gilbert (LLG) equation

2 $$\begin{aligned} \dot{{\mathbf {m}}} = -\gamma {\mathbf {m}}\times {\mathbf {H}}_{\text {eff}} +\alpha {\mathbf {m}}\times \dot{{\mathbf {m}}}, \end{aligned}$$

being $\gamma$ the gyromagnetic ratio, $\alpha$ the phenomenological Gilbert damping and ${\mathbf {H}}_{\text {eff}}$ is the effective field defined as $\mu _0{\mathbf {H}}_{\text {eff}}=-\delta E_m[{\mathbf {M}}]/\delta {\mathbf {M}}$, being $\delta /\delta {\mathbf {M}}$ the variational derivative with respect to the magnetization ${\mathbf {M}}$ and the magnetic energy $E_m$ that now considers the additional Zeeman term coming from the application of the microwave field. The equilibrium configuration is excited with a small magnetic field of the form^42^ $h(t)= h_0\text {Sinc}\left[ 2\pi f_{\text {max}}(t-t_0)\right]$, where $h_0=1$ mT, $f_{\text {max}}=80$ GHz, and $t_0=1$ ns. The amplitude of this pulse must be small enough to keep the system in the linear response regime. The temporal evolution of the magnetization under the action of the exciting field is collected for 4 ns recording the magnetization configuration at uniform time intervals of 1 ps allowing a spectral resolution of 0.25 GHz. Then, the small exciting magnetic field *h*(*t*) and the magnetization distribution *M*(*r*, *t*) are transformed to the frequency domain $[h(\omega ), M(\omega )]$ using the Fast Fourier Transform (FFT) method. The dynamic susceptibility, which corresponds to the imaginary part of the magnetic susceptibility, is calculated by dividing the Fourier transform of the response $M(\omega )$ by the Fourier transform of the excitation $h(\omega )$. Finally, in order to confirm the origin of the resonant peaks, we can reconstruct the spatial profiles of the resonant modes by calculating the temporal Fourier image for each site as ${\tilde{m}}(r_{ijk},f_n)=\text {DFT}_t\left( m(r_{ijk},t)\right)$, where $\hbox {DFT}_t$ is the Discrete-time Fourier Transform, the subscript *ijk* corresponds to the spatial coordinates *x*, *y*, *z* of each cell, and the subscript *n* indicates the position of the frequency in the power spectra. These images are essentially the profiles of the magnetization for any frequency.

## Results and discussion

**Figure 3:**
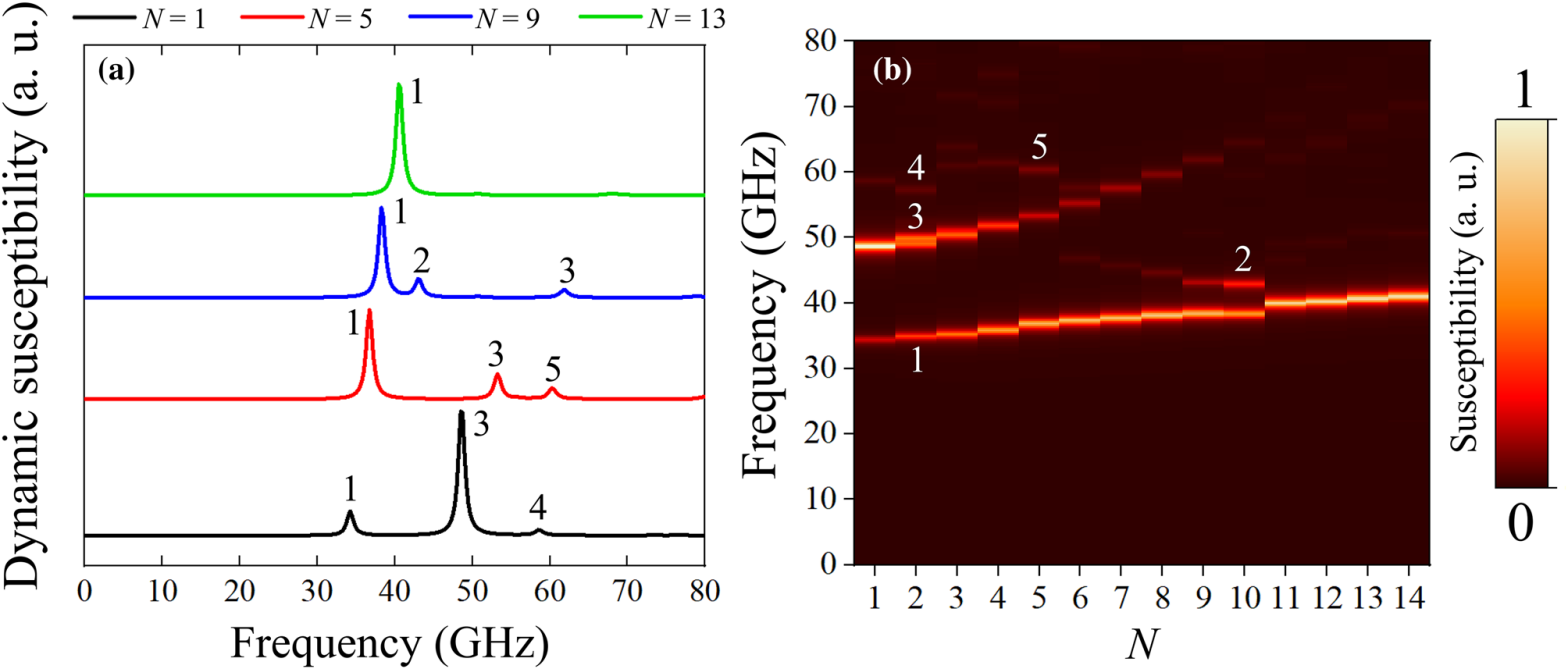
(**a**) Dynamic susceptibility for the cases $N = 1, 5, 9,$ and 13. (**b**) Frequency of the resonance modes plotted as a function of the number of skyrmions hosted in the nanodot. The color bar represents the amplitude of the *z*-component of the absorption spectrum.

Once the magnetic pulse is applied in the *x*-direction, the spin-wave modes are activated with a characteristic frequency and amplitude. Figure 3 shows the frequency and amplitude of these resonance modes as a function of the number of skyrmions hosted in the cluster structure. Figure 3a shows with black, red, blue, and green lines, the dynamic susceptibility for the cases $N=1, 5, 9$, and 13, respectively. It can be clearly seen five distinct modes, which we called *mode 1*, *mode 2*, *mode 3*, *mode 4*, and *mode 5* and they have been marked on each resonant peak. Note that, although modes 2 and 4 might seem the same one, we have confirmed that they have different origin (see Fig. S5 in the supplementary material).

**Figure 4:**
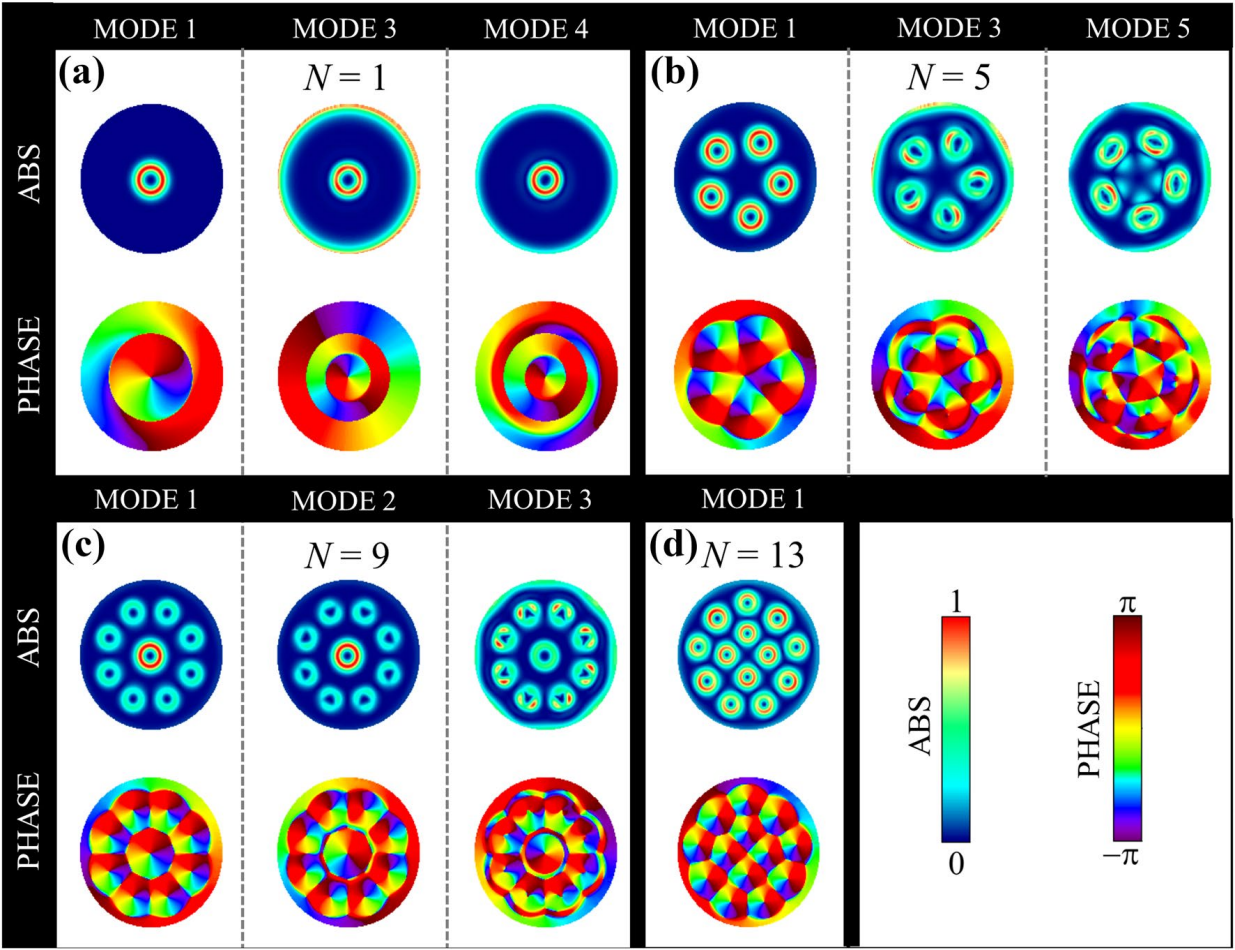
*z*-component and spectral phase of the spatial distribution of the dynamic susceptibility for each resonance frequency of the skyrmion cluster when a magnetic pulse was applied along the *x*-axis for the cases (**a**) $N=1$, (**b**) $N = 5$, (**c**) $N = 9$, and (**d**) $N=13$. In the upper row the bright part (red) reflects high spin precession amplitude and the dark part (blue) corresponds to zero amplitude, while the bottom row represents the phase at which the spins rotate.

Note that the amplitude of the dynamic susceptibility is related to the excited magnetic volume at a certain frequency. Therefore, larger amplitudes are related to a significant magnetic volume coherently excited, while smaller amplitudes are expected to correspond to smaller magnetic volume coherently excited. This can be confirmed when examining the spatial distribution of the reported modes (see Fig. 4 and “Results and discussion” below). The results presented in Fig. 3a suggest the possibility of exciting specific modes according to the number of skyrmions stabilized in the cluster structure. In Fig. 3b, we show the resonance frequency for the entire range of *N* studied here. We can identify basically four scenarios: the first one, for $N<3$, three possible modes could be activated, namely, *modes 1, 3*, and *4*. A distinct scenario occurs for $N=3, 4$ and 5, where it is only possible to excite *modes 1, 3*, and *5*. Next, in the range $5<N<11$ it is possible to excite *modes 1, 2* and *3* according to the resonance frequency. Finally, for *N* greater than $N=11$ only *mode 1* can be excited. It is important to notice that *mode 1* is always the lowest energy mode for our specific choice of magnetic parameters and geometry, while modes 2, 3 and 4 corresponds to higher energy modes. The question that emerges now is which is the nature of the reported modes.

To elucidate the origin of the resonance modes as well as the associated magnetization texture dynamic behind of them, in Fig. 4 we show the *z*-component of the spatial distribution of the dynamic susceptibility for each resonance frequency of the skyrmion cluster distributed according to selected number of skyrmions *N*, and its corresponding spectral phase. The color scale for the upper row in each panel corresponds to the amplitude of spin fluctuations, being red the highest spin amplitude and blue zero amplitude. Similarly, the color scale for the lower row in each panel displays the spectral phase of each mode varying between $\pi$ (darkest red) and $-\pi$ (violet). We start by analyzing the case with $N=1$, which corresponds to an isolated skyrmion, as depicted in Fig. 4a. As can be seen, only modes *1, 3,* and *4* are activated, while modes *2* and *5* cannot be excited. Interestingly, *mode 1* is activated mainly due to the high precession of spins located around the nanodot center, as depicted by the red color around it. This is consistent with the fact that the skyrmion DW acts as a propagation channel of spin waves. Note that the spectral phase reveals a CCW movement of the skyrmion center around the nanodot center. On the other hand, *mode 3* originates from smaller perturbations at the skyrmion DW but also from the characteristic ring-like spin distribution that emerges at the edge of the nanodot. In the same way, *mode 4* has a similar origin as the *mode 3*, but with a smaller spin amplitude at the nanodot edge. In all cases, the spectral phase evidences the presence of radial nodes (see also Fig. S7 in supplementary material), and it is winding $2\pi$ around the center. Thus, we conclude that all reported modes for $N = 1$ correspond to azimuthal-like spin-wave modes emerging from the interaction between a periodic CCW gyration of the skyrmion center and the spins located at the nanodot edge gyrating as a whole.

Next, we analyze the case $N=5$ where the system is subjected to the skyrmion–skyrmion interaction. In this case, *mode 1* is also characterized by the excitation of spins located mainly at the center of each skyrmion in a similar way as the lowest energy mode for the case $N=1$. Note that, in the case $N=5$, the five skyrmions embedded in the cluster structure share the same spin spatial distribution, evidencing a large spin amplitude around each skyrmion center. The spectral phase shows that the five skyrmions coherently gyrate around their centers. Therefore, this case correspond to the spin-waves separately localized at the skyrmion DWs. The next higher frequency mode corresponds to the *mode 3* characterized by the rotation of the skyrmions around their respective centers, and by the spin fluctuation at the nanodot edge. Note that, distinct from the isolated case, here the skyrmions are rotating around their centers with a relative phase between them as can be seen from the spectral phase diagram. Importantly, the *trajectory* followed by each skyrmion is no longer circular, evidencing that skyrmion–skyrmion interaction becomes progressively more dominant. The ring-like spin distribution at the nanodot edge also suggests the presence of azimuthal spin-wave modes. Interestingly, the *mode 5* is activated here, which according to Fig. 4b, is the highest energy mode. In this case the five skyrmions gyrate with a non-regular amplitude (see the oval shape of the spin amplitude), but also, the center region is excited as well due to changes of the magnetization field because of the high frequency gyration of each skyrmion. This complex behavior is also depicted in the spectral phase diagram, where for every skyrmion location, the phase winds $2\pi$ around their center, but with a larger spatial extension (for comparison, see the regular case *mode 1*).

The following case corresponds to $N=9$ as depicted in Fig. 4c. This is a special case because the symmetry of the system pushes to keeping one skyrmion at the nanodot center while the rest of them lie uniformly distributed around it. In this case, the lowest energy mode (*mode 1*) also corresponds to spin-waves propagating along the skyrmion DWs. However, the highest spin amplitude corresponds to the central skyrmion, just like the isolated case $N=1$. Nevertheless, the nine skyrmions gyrate around their center with the same phase and the presence of pseudo-radial nodes allows us to conclude that this is an azimuthal mode. The next higher energy mode is the *mode 2* that, according to the spectral amplitude, is pretty like *mode 1*. In fact, the central skyrmion is equally excited as the above case (but with a different frequency), gyrating thus around the nanodisk center, but the surrounding skyrmions oscillate around their centers with a non-regular shape. Interestingly, a crucial difference with the *mode 1*, is that in this case all surrounding skyrmions oscillate with the same phase, but the central one oscillates with a a different phase than theirs. Therefore, we conclude that this mode also corresponds to an azimuthal mode but with a different phase between the central and the surrounding skyrmions. Next, the *mode 3* is also an azimuthal spin-wave mode characterized by the ring-like spin distribution at the nanodot edge. Specifically, the highest spin amplitude corresponds now to the surrounding skyrmions, which also oscillate with a non-regular amplitude, as characterized by the oval shape in the spectral phase. Furthermore, all the skyrmions gyrate around their center with a relative phase between them. Interestingly, these modes are exactly the three lowest energy modes, which suggest that the system prefers to firstly excite with high spin amplitude the skyrmions at the center and then the surrounding ones, if the nanodot center is occupied with a skyrmion. This is the case for $N = 6, 7, 8, 9$ and 10, which are capable to excite the $\textit{mode 2}$, as evidenced in Fig. 4b. This behavior can be understood in terms of the skyrmion–skyrmion interaction between the central and surrounding skyrmions due to the proximity between them (see also Supplementary information for a further discussion).

Finally, the case $N=13$ is depicted in Fig. 4d, where only *mode 1* is activated and almost all skyrmions precess with the same spin amplitude. The spectral phase shows they all oscillate with the same phase. Therefore, it can be described as a separated azimuthal mode for each skyrmion hosted in the nanodot. Thus, we have described a system whose main feature corresponds to the ability of *turn on* or *turn off* certain spin-wave modes according to the number of skyrmions *N* stabilized in the cluster structure. This behavior mimics the operation of a *magnonic key*, which can be *opened* or *closed* according to the number of skyrmions and the frequency at which the distinct modes are activated. The operating principle of this potential device demands to accurately manage the number *N* of skyrmions hosted in the cluster structure. Recently was established through micromagnetic simulations^16,37^ that, by applying an out-of-plane magnetic pulse, it is possible to freely choose the number of skyrmions in the cluster structure by only knowing the characteristic frequency at which a specific spin-wave mode is activated. Therefore, by combining such a method with the proposed one here, the operating principle of the *magnonic key* is fully satisfied and might emerge as an alternative for microwave spintronic devices.

## Conclusions

We have studied the spin-wave modes that appears when applying an in-plane magnetic field on a skyrmion cluster structure, finding basically five resonant peaks. These resonant modes are shown to be dependent on the number of skyrmions hosted in the nanostructure. Note that, different from previous studies *mode 1* is the lowest energy mode and do not correspond to the gyrotropic one. Instead, our system describes azimuthal spin-wave modes for all cases. The next higher energy mode (*mode 2*) is an azimuthal mode too, but now with a relative phase between the central and the surrounding skyrmions; therefore, the *mode 2* is only activated in the presence of a central skyrmion together to the strong skyrmion–skyrmion interaction between them, and whose main feature is to provide such a phase difference. A very important point is that all the azimuthal spin wave modes found here cannot be classified in terms of the usual (*n*, *m*) pairs numbers^43,44^ since we have a multi-skyrmion state and the sense of *nodes* is lost. Nevertheless, we can identify that the skyrmions in our system always perform a CCW gyration, thus, the difference between the modes is only attributable to a distinct radial quantization. This is consequence of the magnetic parameters used here, which do not allow larger skyrmions radii. It is important to point out that the reported behavior of the frequency modes corresponds to a feature of the specific magnetic parameters used here. Particularly, eventual modifications on the DMI or magnetic anisotropy constant would change the skyrmions radius, and then modifying the frequency modes. Finally, we proposed our system as a versatile *magnonic key* due to the ability of *turn on* or *turn off* desired spin-waves modes by only controlling the number of skyrmion in the system. This proposal might be well accomplished with the current experimental techniques subjected to a low temperature regime.

## Supplementary Information

Supplementary Information.
