# Supplementary material for: Magnonic key based on skyrmion clusters
Source: Sci Rep. 2021 Nov 26;11:23010. doi: 10.1038/s41598-021-02285-0 (PMC8626437; doi:10.1038/s41598-021-02285-0)
Supplement: Supplementary file 1 — Supplementary Information. [file 41598_2021_2285_MOESM1_ESM.pdf]

SUPPLEMENTARY INFORMATION

Magnonic key based on skyrmion clusters

E. Saavedra and J. Escrig

Departamento de Física, Universidad de Santiago de Chile (USACH), 9170124 Santiago, Chile and Center for the Development of Nanoscience and Nanotechnology (CEDENNA), 9170124 Santiago, Chile

F. Tejo

Instituto de Ciencia de Materiales de Madrid, CSIC, Cantoblanco, 28049 Madrid, Spain

N. Vidal-Silva

Departamento de Ciencias Físicas, Universidad de La Frontera, Casilla 54-D, 4811186 Temuco, Chile.

(Dated: October 8, 2021)

I. METASTABLE MAGNETIC STATES FOR DIFFERENT NUMBER OF SKYRMIONS STABILIZED IN THE CLUSTER STRUCTURE

Here we show the distinct numbers of skyrmions stabilized in the nanodot with $R = 75$ nm. It

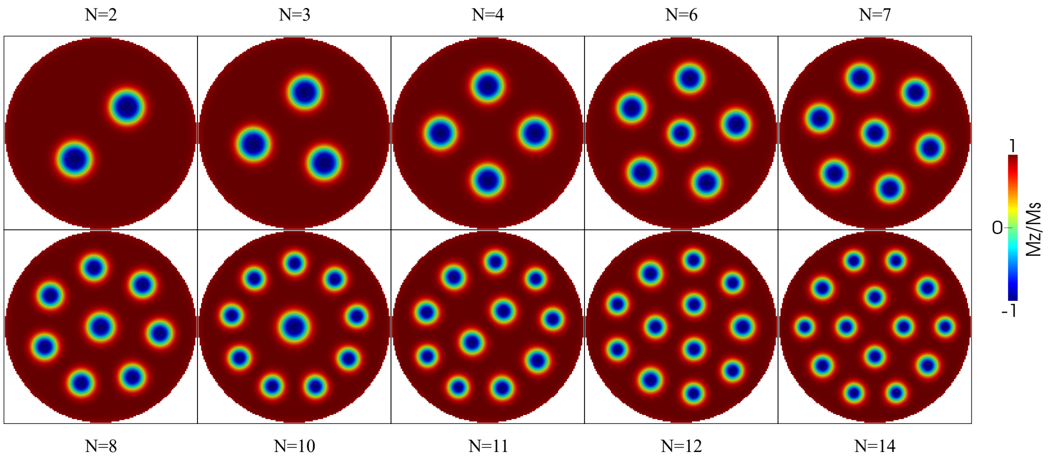

FIG. S1. Metastable magnetic states obtained from the minimization energy procedure for the cases $N = 2, 3, 4, 6, 7, 8, 10, 11, 12$ and 14.

is important to mention that, for the chosen magnetic parameters, $N = 14$ is the maximum value of N we can reach. When intending to stabilize a larger number of skyrmions, the metastable states obtained through the minimization energy procedure converges into a ferromagnetic homogeneous state. Note that, as stated in the main text, when $5 < N < 11$ the system accommodates a skyrmion at the central region.

II. DYNAMIC SUSCEPTIBILITY AND RESONANCE FREQUENCY AS A FUNCTION OF N FOR DISTINCT VALUES OF R

In this section, we show the dynamical response for different values of R. It is possible to see that, in general, the behavior reported for the case $R = 75$ nm in the main text holds even for different values of R. However, we note that *mode 2* is progressively attenuated as R increases. We attribute it to the weaker skyrmion-skyrmion interaction that the central skyrmion feels due to the larger inter-skyrmions distance.

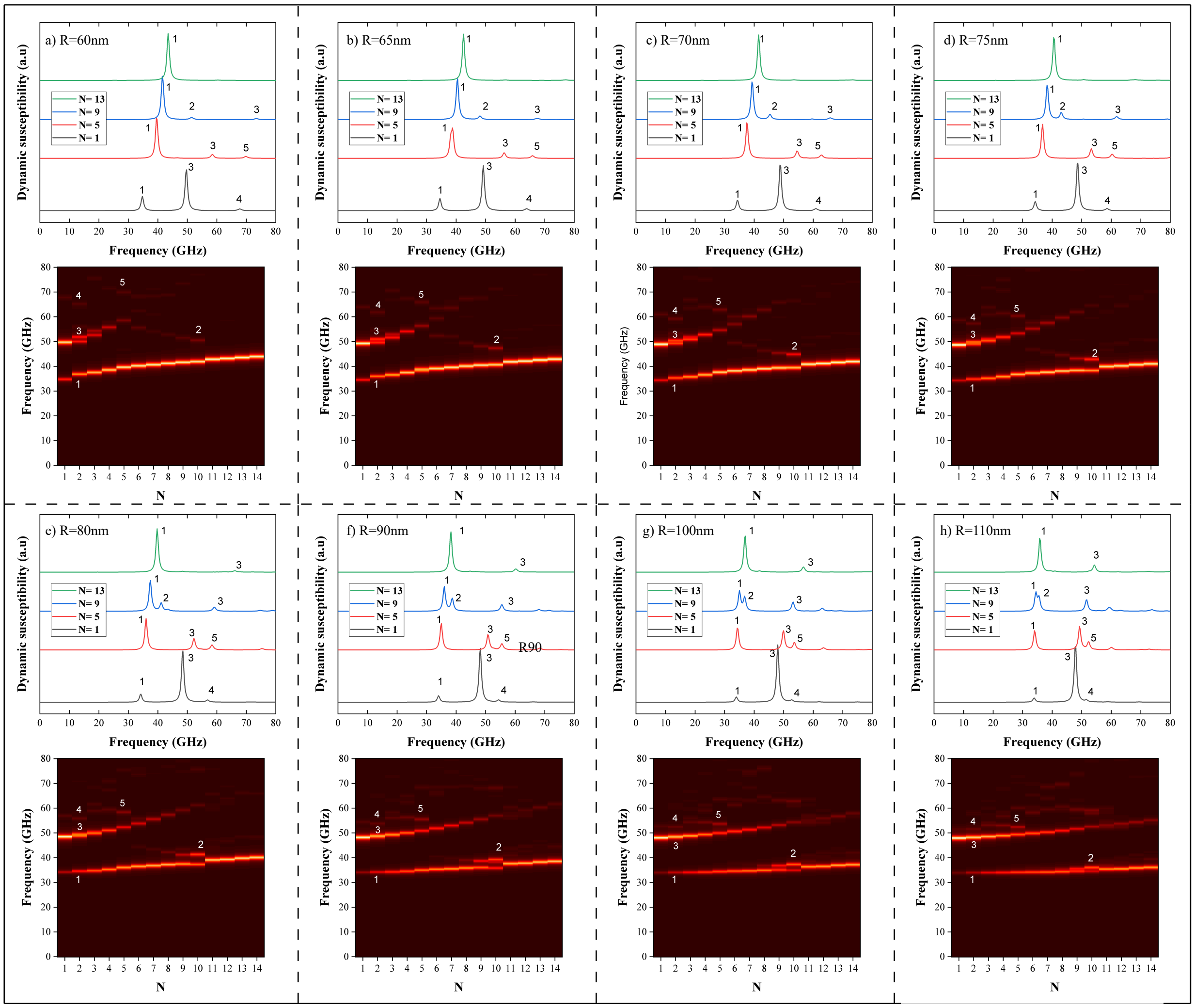

FIG. S2. Dynamic susceptibility for selected values of N and resonance requency as a function of N for a) $R = 60$ nm, b) $R = 65$ nm, c) $R = 70$ nm, d) $R = 75$ nm, e) $R = 80$ nm, f) $R = 90$ nm, g) $R = 100$ nm, and h) $R = 130$ nm.

III. SPECTRAL AMPLITUDE AND PHASE OF RESONANCE MODES FOR DIFFERENT VALUES OF N WITH $R = 75$ NM

Here we show the spectral amplitude and phase for the remaining values of N not listed in the main text. In the absence of a central skyrmion, the *mode 1* is always a gyrotropic one and the next higher energy modes are azimuthal-like (see cases $N = 3$ and 14). However, in the presence of a central skyrmion both *mode 1* and *mode 2* are pseudo-gyrotropic, while the next higher energy mode (*mode 3*) is azimuthal. This is explained in terms of the skyrmion-skyrmion interaction

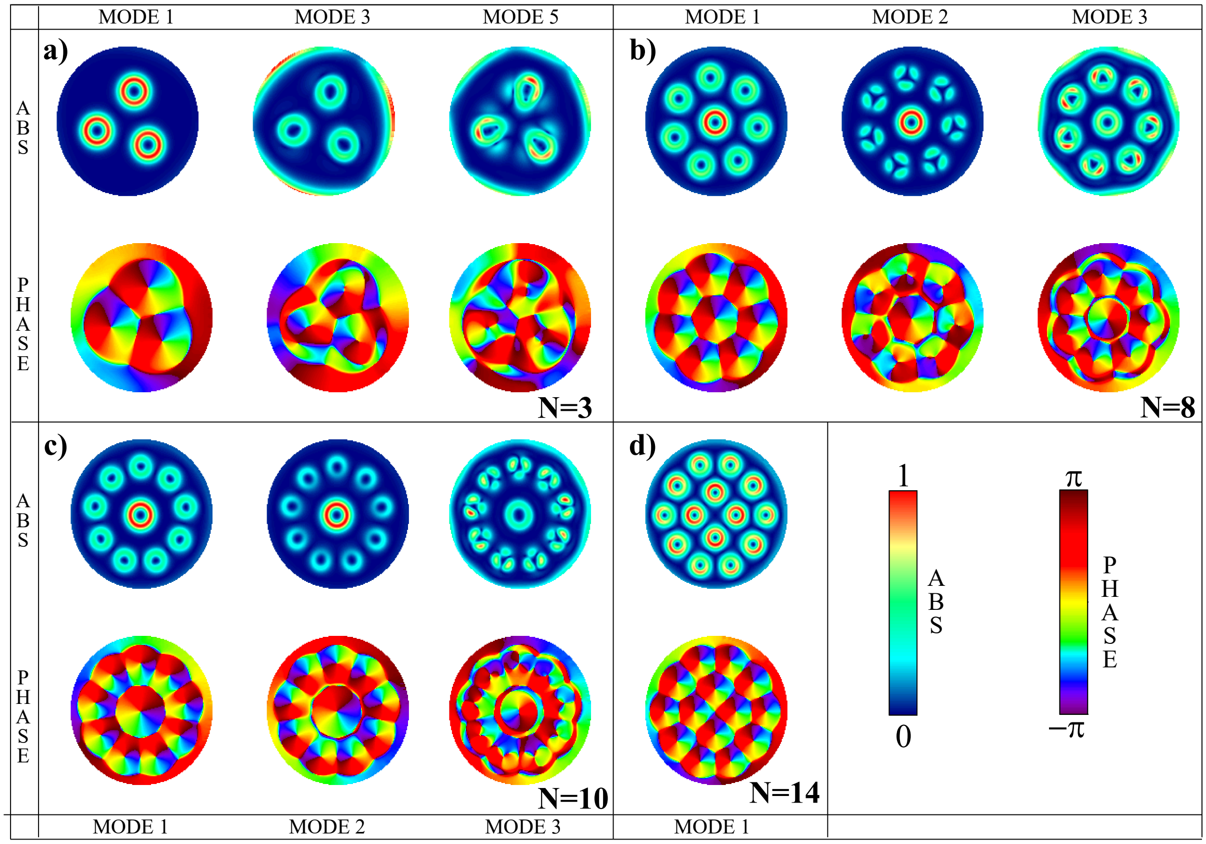

FIG. S3. $z-$component and spectral phase of the spatial distribution of the dynamic susceptibility for each resonance frequency of the skyrmion cluster when a magnetic pulse was applied along the $x-$axis for the cases a) $N = 3$, b) $N = 8$, c) $N = 10$, and d) $N = 14$

between the central and surrounding skyrmions.

IV. COMPARISSON OF THE SKYRMION RADIUS BETWEEN CENTRAL AND SURROUNDING SKYRMIONS

In order to explain the presence of *mode 2* at the values of N where the system stabilizes a central skyrmion, and the tendency to disappear as long as R increases, we have measured the radius of both skyrmions (central and surrounding ones) for different values of the nanodot size R. As can be seen, when R increases, the radius of both skyrmions progressively becomes comparable between them, which is an indicator of a weaker skyrmion-skyrmion interaction and a larger distance of separation. Therefore, as R is increased, such an interaction decreases, and the system

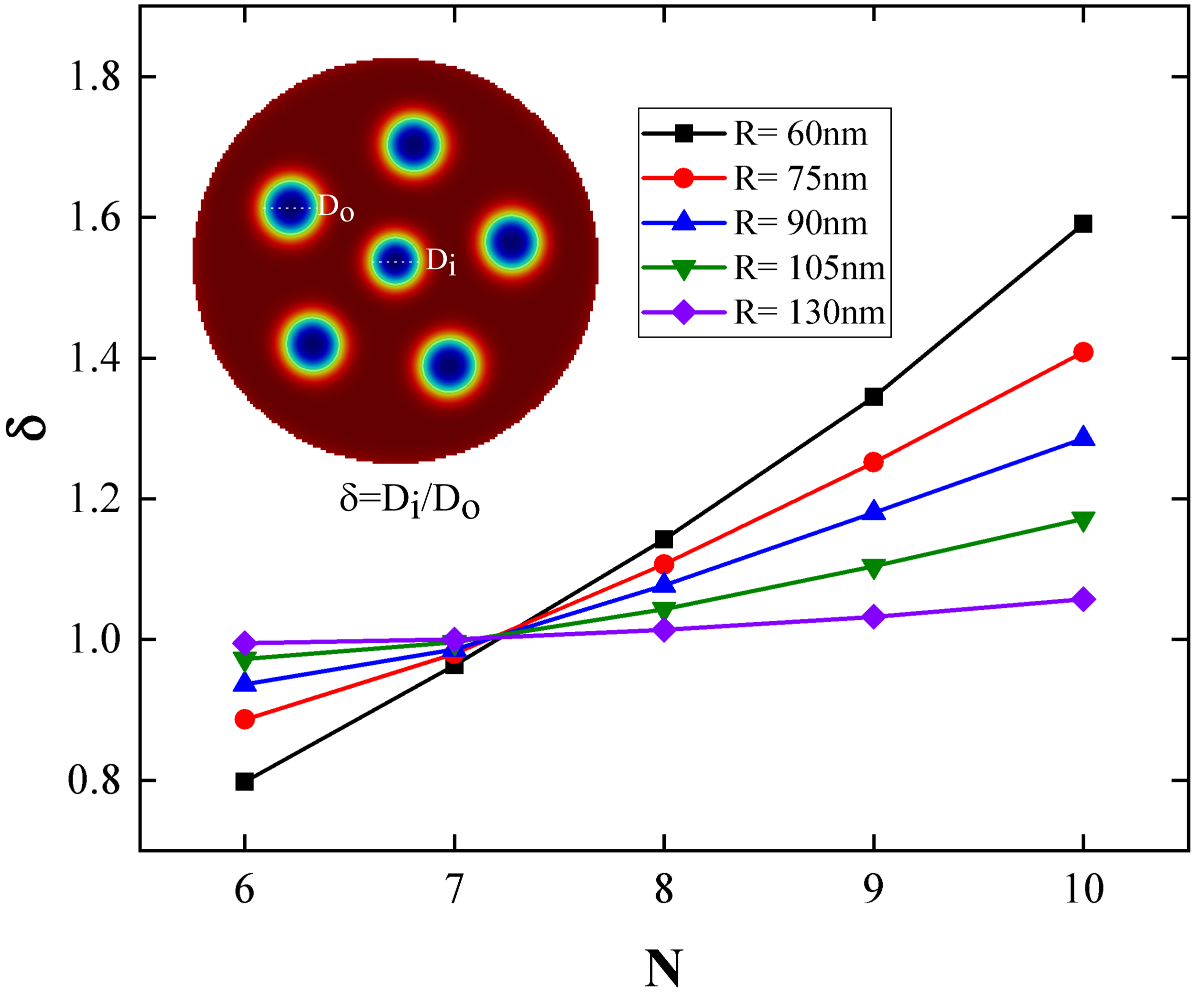

FIG. S4. Ratio of the inner and outer skyrmions radius as a function of N for selected sizes of nanodot R.

tends to excite the central skyrmion with the same phase as the surrounding ones, annihilating thus the *mode 2*.

V. ORIGIN OF DISTINCT REPORTED MODES

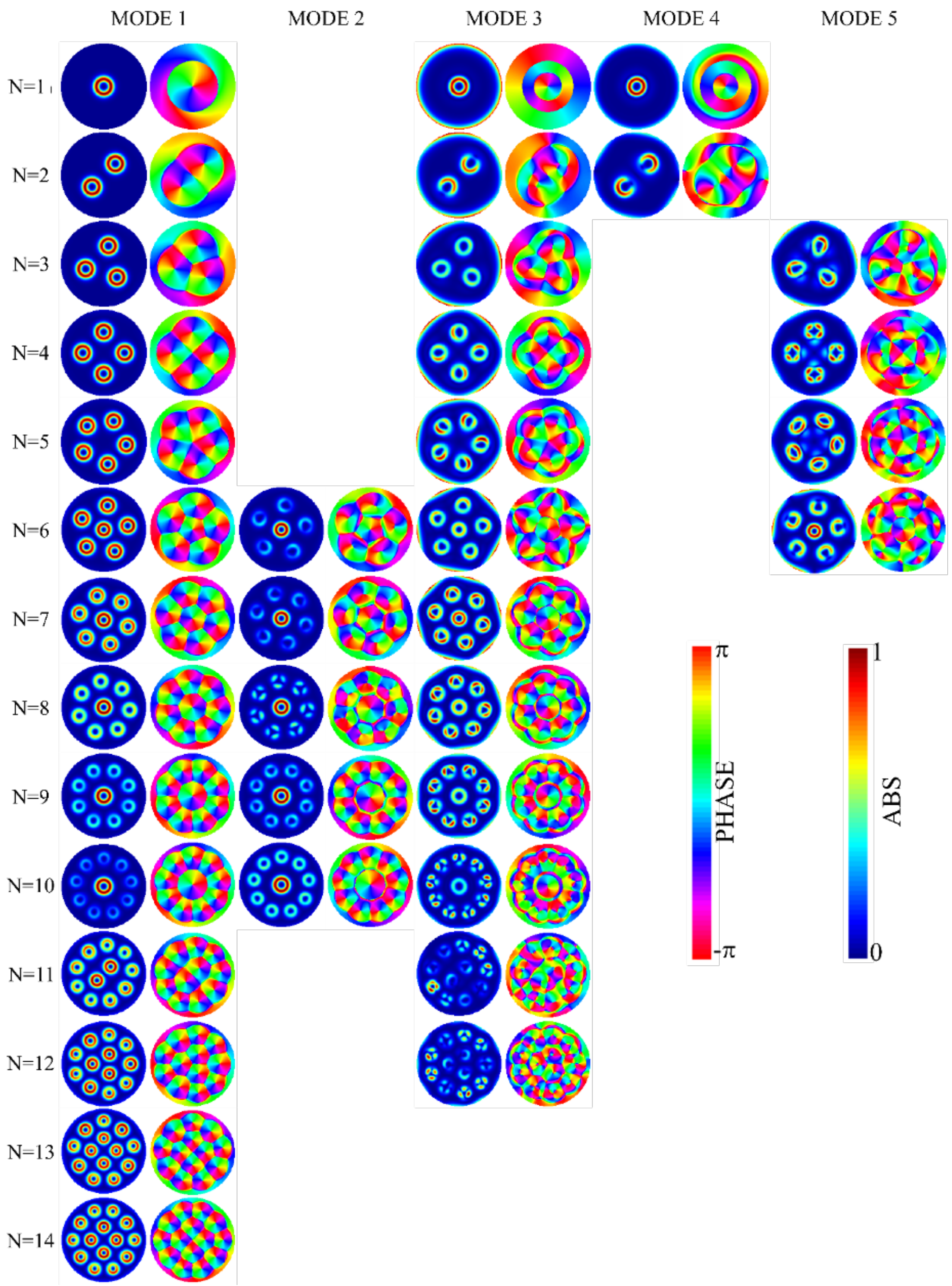

FIG. S5. $z-$component and spectral phase of the spatial distribution of the dynamic susceptibility for each resonance frequency of the skyrmion cluster when a magnetic pulse was applied along the $x-$axis for distinct values of N.

VI. DEPENDENCE OF THE SPIN-WAVE MODES WITH THE α PARAMETER

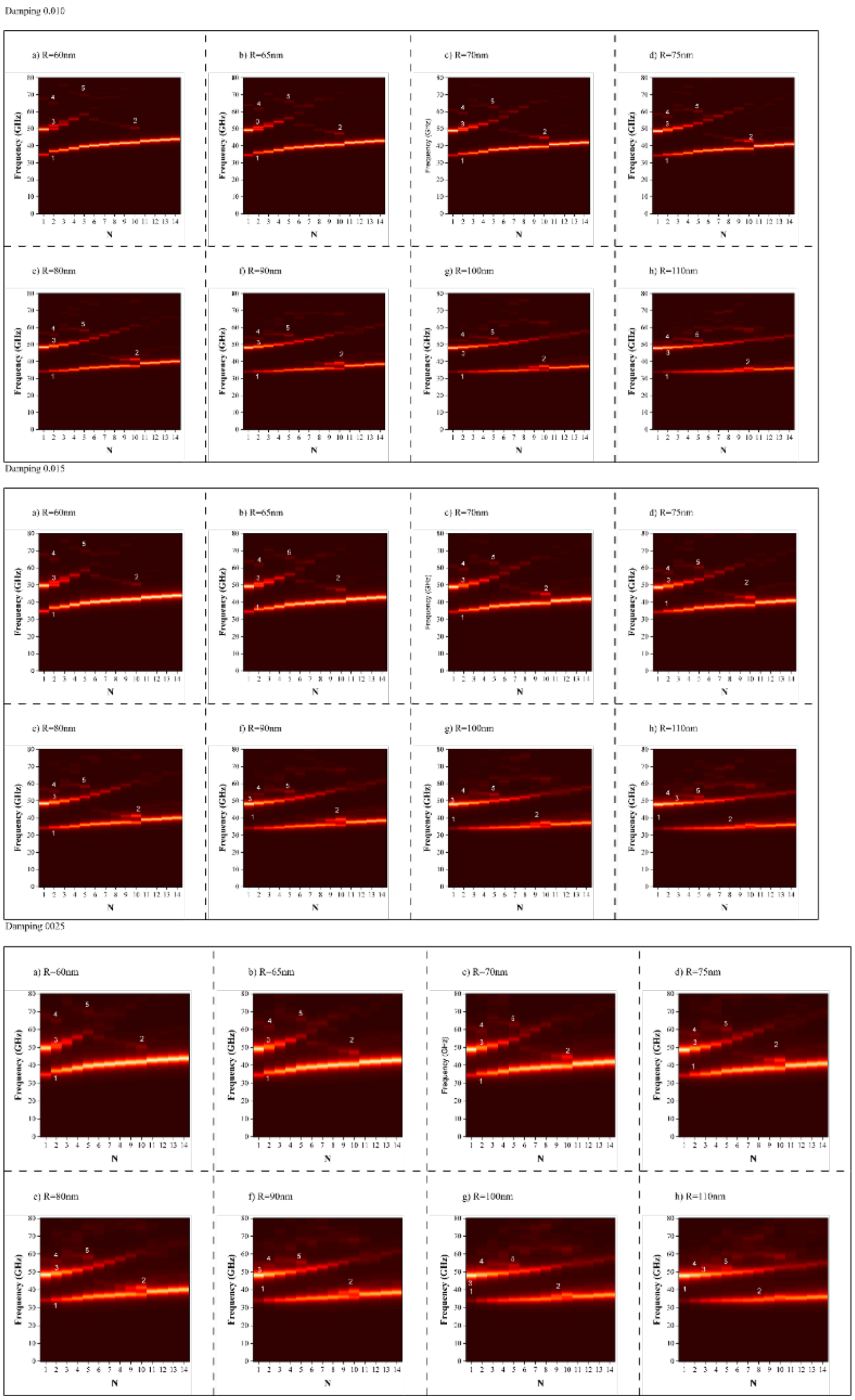

FIG. S6. Frequency of the resonance modes plotted as a function of the number of skyrmions hosted in the nanodot for selected nanodot radii and Gilbert damping values $\alpha = 0.01$, $\alpha = 0.015$ and $\alpha = 0.025$

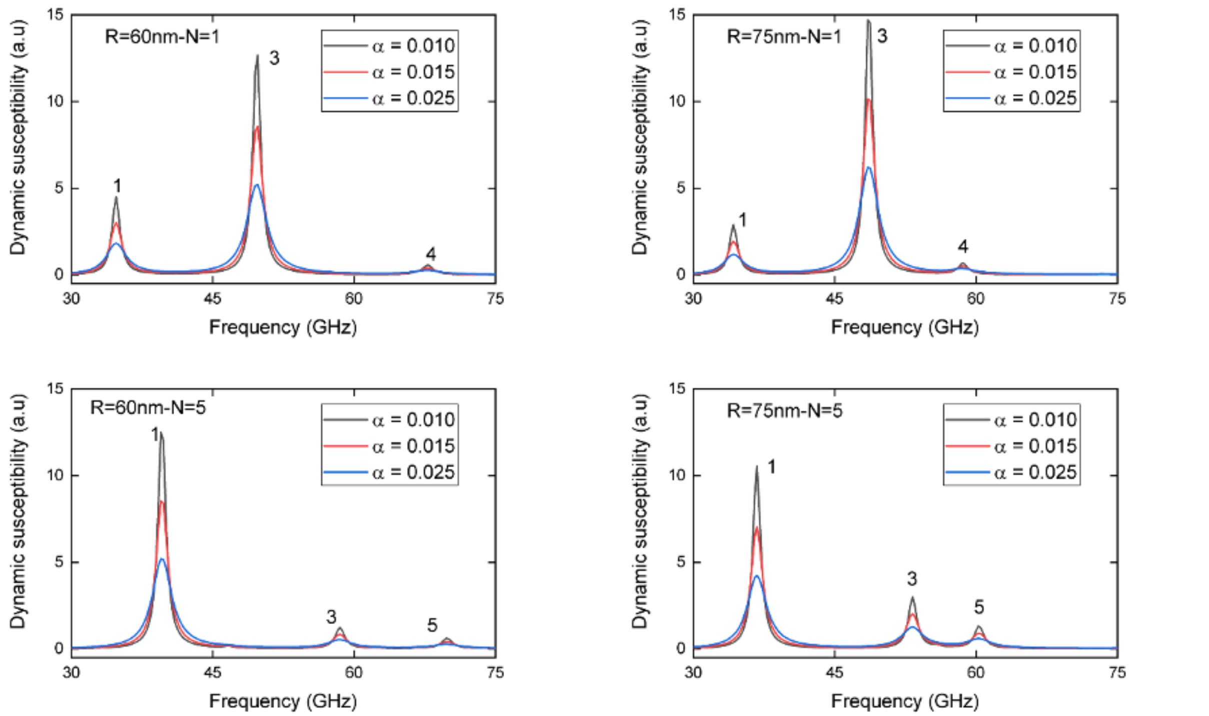

FIG. S7. Dynamic susceptibility for the cases N = 1 and 5, with distinct values of the nanodot radii and the Gilbert damping

VII. ANGULAR DEPENDENCE OF THE DYNAMIC SUSCEPTIBILITY

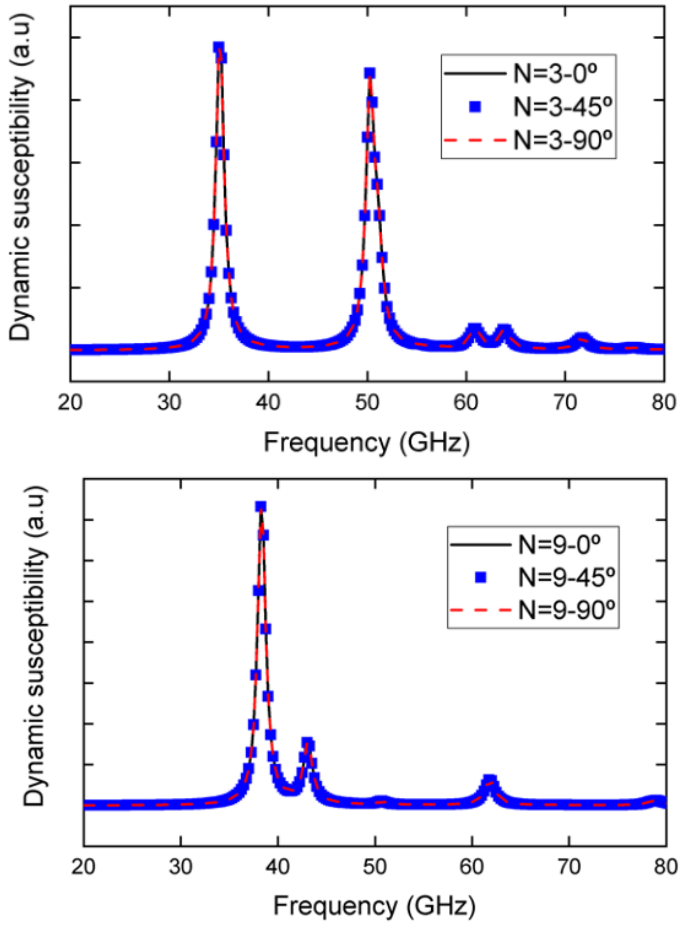

FIG. S8. Dynamic susceptibility for the system under the magnetic pulse applied in distinct angles.

VIII. RADIAL NODES FOR SELECTED CASES OF THE SYSTEM STUDIED.

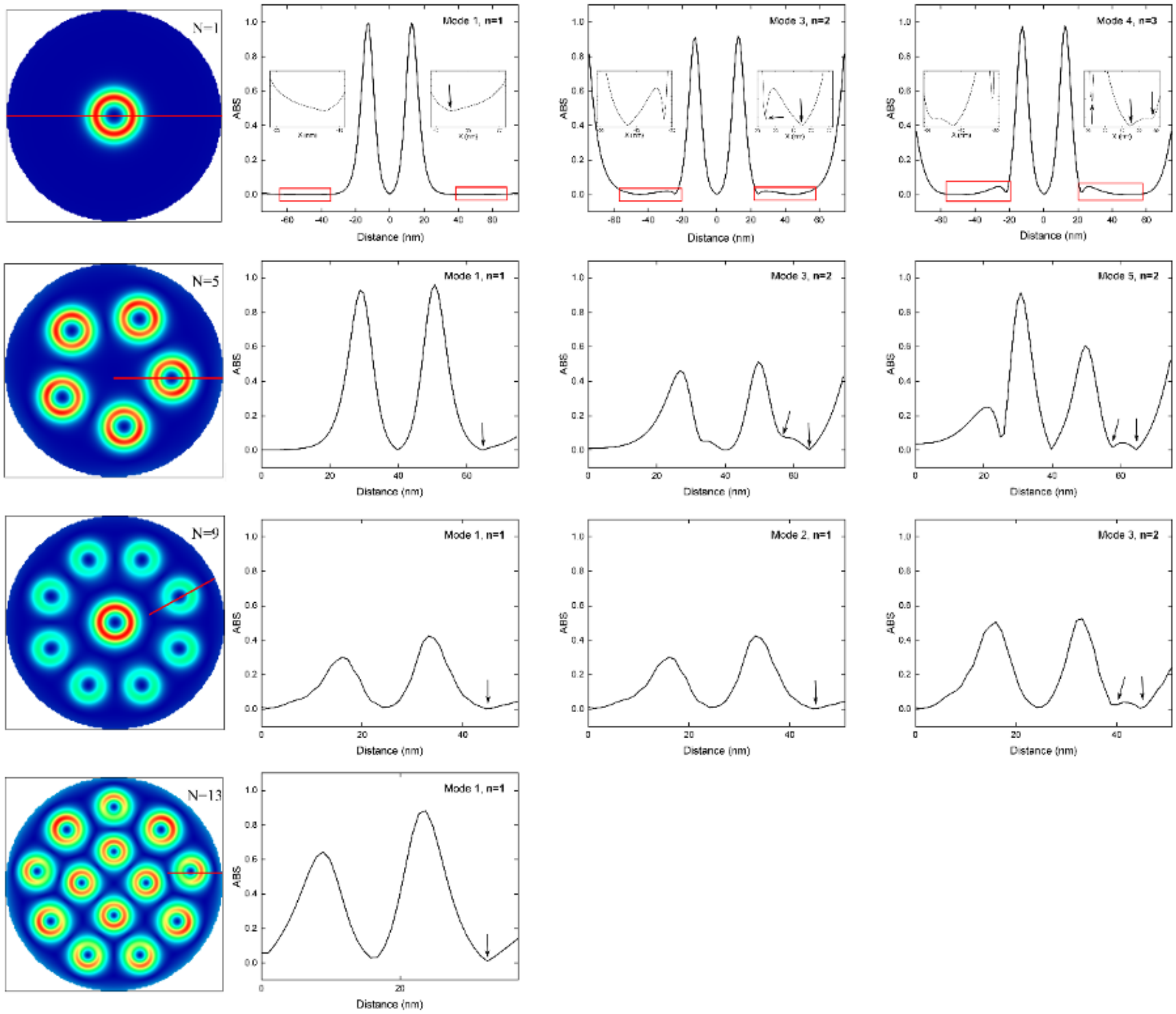

FIG. S9. Identification of the radial nodes for selected modes and distinct N values. The transverse red line crossing a given skyrmion in the left panel corresponds to the radial profiles shown in the right panels for distinct modes.
